# Functions and strategies for enhancing zinc availability in plants for sustainable agriculture

**DOI:** 10.3389/fpls.2022.1033092

**Published:** 2022-10-07

**Authors:** Muhammad Hamzah Saleem, Kamal Usman, Muhammad Rizwan, Hareb Al Jabri, Mohammed Alsafran

**Affiliations:** ^1^ Office of Academic Research, Qatar University, Doha, Qatar; ^2^ Agricultural Research Station, Office of VP for Research and Graduate Studies, Qatar University, Doha, Qatar; ^3^ Center for Sustainable Development (CSD), College of Arts and Sciences, Qatar University, Doha, Qatar; ^4^ Department of Biological and Environmental Sciences, College of Arts and Sciences, Qatar University, Doha, Qatar; ^5^ Central Laboratories Unit (CLU), Office of VP for Research and Graduate Studies, Qatar University, Doha, Qatar

**Keywords:** zinc deficiency, ion uptake and gene transporters, organic ligands, root architecture, biostimulators, nano-fertilizers

## Abstract

Zinc (Zn), which is regarded as a crucial micronutrient for plants, and is considered to be a vital micronutrient for plants. Zn has a significant role in the biochemistry and metabolism of plants owing to its significance and toxicity for biological systems at specific Zn concentrations, i.e., insufficient or harmful above the optimal range. It contributes to several cellular and physiological activities of plants and promotes plant growth, development, and yield. Zn is an important structural, enzymatic, and regulatory component of many proteins and enzymes. Consequently, it is essential to understand the interplay and chemistry of Zn in soil, its absorption, transport, and the response of plants to Zn deficiency, as well as to develop sustainable strategies for Zn deficiency in plants. Zn deficiency appears to be a widespread and prevalent issue in crops across the world, resulting in severe production losses that compromise nutritional quality. Considering this, enhancing Zn usage efficiency is the most effective strategy, which entails improving the architecture of the root system, absorption of Zn complexes by organic acids, and Zn uptake and translocation mechanisms in plants. Here, we provide an overview of various biotechnological techniques to improve Zn utilization efficiency and ensure the quality of crop. In light of the current status, an effort has been made to further dissect the absorption, transport, assimilation, function, deficiency, and toxicity symptoms caused by Zn in plants. As a result, we have described the potential information on diverse solutions, such as root structure alteration, the use of biostimulators, and nanomaterials, that may be used efficiently for Zn uptake, thereby assuring sustainable agriculture.

## Introduction

Zinc (Zn) is an important micronutrient for plants since it is involved in many key cellular functions such as metabolic and physiological processes, enzyme activation, and ion homeostasis (Yang et al., 2020; Alsafran et al., 2022). Living organisms require Zn as a trace element at minimal levels for regular metabolic activities (Sturikova et al., 2018). Additionally, Zn is a crucial nutrient for plants and is involved in a number of their bio-physicochemical responses (Noman et al., 2019; Zaheer et al., 2022). Zn has an important role as a constituent of over 300 enzymes from all six enzyme classes. This is the only element found across all six enzyme classes (lyases, transferases, hydrolases, isomerases, oxidoreductases, and ligases). Zn influences the activity, structural integrity, and folding of numerous proteins as a fundamental or catalytic enzyme (Castillo-González et al., 2018; Zaheer et al., 2020b). In addition to its role as a key factor for the structural integrity of ribosomes, Zn plays a number of other important bio-physicochemical roles in plants, including gene regulation and activation, protein synthesis, involvement in carbohydrate metabolisms, and morphological and anatomical participation in bio-membranes (Hafeez et al., 2013; Zaheer et al., 2020a). Additionally, the interaction between Zn phospholipids and sulfhydryl clusters of membrane proteins enhances membrane stability (Kathpalia and Bhatla, 2018; Kareem et al., 2022).

**Figure 1:**
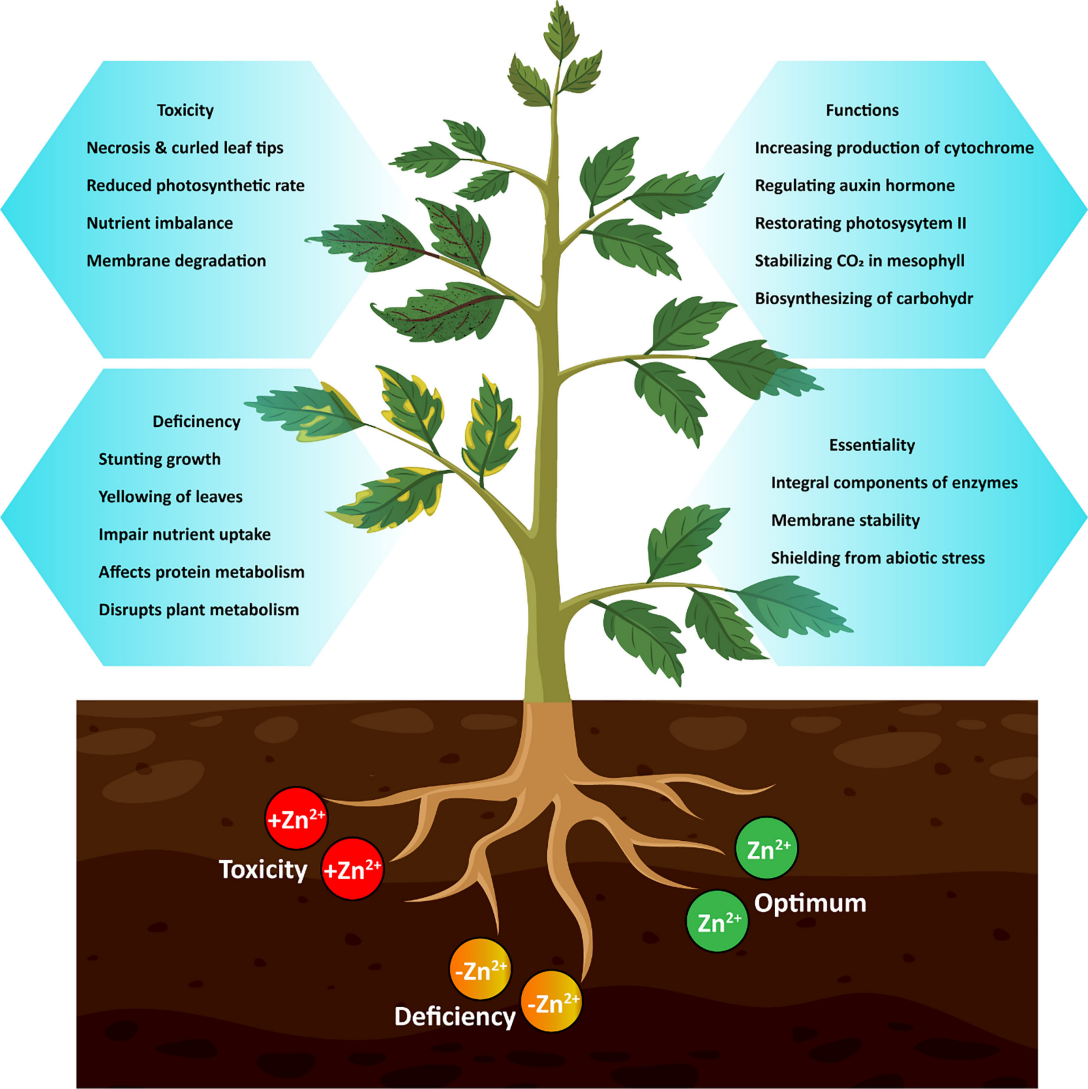
Functions, deficiency and toxicity effect of Zn on plants.

The Zn-binding proteins are estimated to make up around 6% of the prokaryotic proteome and approximately 9% of the eukaryotic proteome (Andreini et al., 2006). The Zn-containing proteins in plants include enzymes such as carbonate dehydratases, aldehyde dehydrogenases, Zn/copper (Cu) superoxide dismutase (SOD), and Zn-finger DNA-binding proteins (Sofo et al., 2018; Al Jabri et al., 2022). Superoxide radicals are neutralized by Zn-Cu-SOD, therefore shielding membrane proteins and lipids from oxidation. This nutrient is needed for seed development and increases the production of cytochrome (Karthika et al., 2018; Alatawi et al., 2022). Moreover, Zn is important for a number of physiological functions in plants, such as regulating hormones (like the production of tryptophan, which is a precursor of indole acetic acid) and sending signals through mitogen-activated protein kinases (Bhantana et al., 2021; Kaur and Garg, 2021; Ahmad et al., 2022). The plant-specific functions that depend on Zn availability include regulation of auxin, restoration of photosystem II, stabilization of CO_2_ amount in the mesophyll (Gupta et al., 2016), and other physiological responses as shown in **Figure 1**. Simultaneously, Zn ions can inactivate proteins by attaching to their functional groups or displacing other cations from binding sites, thus reducing a variety of harmful effects on plant cells (Natasha et al., 2022). Therefore, it is crucial for plants to properly absorb, transport, and distribute Zn in their tissues, cells, and intracellular locations to ensure the proper functioning of the plant (Zlobin, 2021).

Despite its importance, Zn deficiency disrupts the basic operations of plant metabolism, causing growth retardation and leaf chlorosis, which can impair nutrient intake and eventually contribute to Zn deficiency in the human diet (Li et al., 2013). Zn deficiency is a serious problem in agricultural soils across the world, due to limited amount of Zn present in soils is available for plant uptake, because the entire Zn is available in structural minerals and absorbed by other soil components (Ali et al., 2022a; Ali et al., 2022c). Therefore, accumulating evidence suggests that exogenous Zn can mitigate the oxidative damage to the cell membrane caused by unfavorable environmental circumstances, such as heavy metal stress (Zhou et al., 2019). On the other hand, Zn toxicity, competes with necessary element absorption, stimulates the generation of reactive oxygen species, and results in heavy metal toxicity (Zhang et al., 2020). Addressing the molecular and biochemical responses of plants and tolerance to Zn deficiency might help to improve Zn use efficiency and Zn accumulation in plants. In light of this, we have compiled a summary of the literature on the physiological and biochemical strategies of plants to Zn deficiency, the roles of transporters and metal-binding molecules in Zn homeostasis. Moreover, the non-traditional methods that can be used for Zn acquisition and thus result in resource conservation, including alterations to root structure, the use of bio-stimulators and nanotechnology. We have also discussed some novel approaches that may play a role in Zn uptake and translocation under Zn deficiency, including the inoculation of arbuscular mycorrhizal fungi and rhizobacteria, the application of organic ligands in combination with nano-fertilizers, and the use of natural and synthetic chelators.

## Zinc deficiency and toxicity symptoms in plants

Zn deficiency is becoming a severe agricultural concern due to its poor availability in arable soils globally, resulting in decreased crop output and nutritional quality (Zeng et al., 2021). Numerous factors negatively affect the available Zn in soils for plants, including low/total Zn contents, high pH >7, low redox potential, prolonged flooding, microbial communities in the rhizosphere, high organic matter, high calcium carbonate and bicarbonate contents, high iron/manganese oxide contents, high exchangeable magnesium/calcium ratio, high sodium and phosphorus availability (Zhang et al., 2012; Moreno-Lora and Delgado, 2020). A decrease in the protein synthesis was observed in the Zn-deficient plants. However, the process was improved by providing Zn to plants which were Zn deficient/lacking in it (Zeng et al., 2021). As Zn is a structural component of ribosomes, Zn deficiency causes the ribosomes to break down, which affects protein metabolism (Castillo-González et al., 2018). According to Hafeez et al. (2013), low Zn levels actually inhibit the synthesis of RNA and proteins since Zn plays a significant role in several enzymes that are essential for protein synthesis, including glutamate dehydrogenase, ATPase, and ribonuclease. Nevertheless, the amount to which Zn deficiency levels inhibit protein and RNA production varies among plant species.

The prominent signs of Zn deficiency (**Figure 1**) in plants are the yellowing of immature leaves, smaller leaf size, and withered stems. However, older leaves often display leaf bronzing, limited growth, rolling, and wilting of leaves under severe Zn deficiency stress (Mattiello et al., 2015; Zhao and Wu, 2017; Xie et al., 2019). Plants developed certain mechanisms against Zn deficiency stress, including changes in the architecture of root system, relationship of arbuscular mycorrhiza symbiosis, production of phyto-siderophores and organic acid anions, and strengthening of defense mechanism against oxidative stress induced by Zn deficiency (Zhao and Wu, 2017). These modifications in plants boost Zn absorption and transport influenced by Zn transporters (Ricachenevsky et al., 2015) and affect the transcription of important Zn-requiring activities (Gong et al., 2020).

Conversely, excessive Zn exposure to plants can lead to a variety of harmful effects in bio-physico-chemical systems (Bankaji et al., 2019; Sidhu et al., 2020). Zn toxicity causes scarcity of other vital nutrients by interfering with phyto-uptake and transport within plants due to comparable ionic radii (Bankaji et al., 2019). Therefore, Zn disrupts photosynthetic activity, stomatal conductance, and numerous other vital metabolic functions. Zn toxicity reduces plant development and structural stability (Chakraborty and Mishra, 2020), and promotes chlorosis of leaves as shown in **Figure 1**. Substantial levels of Zn produce oxidative damage by encouraging the generation of reactive oxygen species, which cause membrane lipid peroxidation and, in severe cases, leads to plant death (Goodarzi et al., 2020). However, plants have developed numerous defensive and restorative mechanisms, including enzymatic and non-enzymatic antioxidant systems, to combat environmental stresses (Chakrabarti and Mukherjee, 2021; Noor et al., 2022).

Most plant species have Zn concentrations of between 30 and 100 µg Zn^2+^ g^−1^ dry weight, indicating that the zinc homeostasis mechanism is not ubiquitous within plants (Van De Mortel et al., 2006). However, some species like Brassicaceae known as Zn hyperaccumulators, acquire more than 10,000 µg Zn^2+^ g^−1^ dry weight without displaying signs of toxicity. Irrespective of this, Zn^2+^ levels above 300 µg Zn^2+^ g^−1^ dry weight are considered detrimental to plants (Bayçu et al., 2017; Sperdouli et al., 2022). Many studies in **Table 1** have demonstrated the effects of Zn on plants. Hence, it is considered that multiple metabolic functions in plants can be inhibited by either a deficiency of Zn^2+^ or a surplus of Zn^2+^ in the soil which can lead to stunted plant growth and decreased photosynthetic activity, inducing chlorosis and necrosis of the leaf (Andresen et al., 2018; Dobrikova et al., 2021).

**Table 1:** Beneficial, deficiency symptoms, and toxic effects of zinc on plants.

Crop	Concentration	Effect	Reference
*Triticum aestivum* L.	10–100 μM ZnSO_4_.7H_2_O	Enhanced photosynthetic ability Stimulating the activities of antioxidant enzymes	(Wei et al., 2022)
*Zea mays* L.	1.5 ppm ZnSO_4_	Enhanced physiological parameters, e.g. relative water content, yield. Chlorophyll content increased in Zn-treated plants than Zn-untreated plants but chlorophyll fluorescence increased only at higher Zn concentration condition.	(Munirah et al., 2015)
*Pisum sativum* L.	0–1% ZnSO_4_	0.5% was best to increase number of pods/plant, weight of pod, number of seeds/pod, weight of seed/pod and yield.	(Borah and Saikia, 2021)
*Vigna unguiculata* L.	50 ppm ZnSO_4_	Highest germination percentage, root length, shoot length, dry weight and uptake of macro as well as micronutrients.	(Basha and Selvaraju, 2015)
*Beta vulgaris L.*	1.2–300 μM ZnSO_4_	Plants developed symptoms of Zn deficiency, including decreases in Zn content, reduced chlorophyll and carotenoid contents, increases in chlorophyll a/b ratios and de-epoxidation of violaxanthin cycle pigments. Decreased photosystem II efficiency	(Sagardoy Calderón et al., 2008)
*Helianthus annuus L.* cv. Jwala Mukhi	0.00065, 0.0065, 0.065, 0.65, 6.5, and 65 mg L^-1^ ZnSO_4_	Foliar symptoms of Zn deficiency appeared on mature middle and old leaves of plants at 0.00065 and 0.0065 mg Zn as interveinal chlorosis and fading of green color from margins and apices. Later, leaf apices of the affected leaves turned necrotic and dry.	(Khurana and Chatterjee, 2001)
*Zea mays* L.	0–3 ppm ZnSO_4_	The net photosynthesis rate decreased.	(Munirah et al., 2015)
*Triticum aestivum* L.	300–1000 μm 7H_2_O.ZnSO_4_	Induced oxidative stress, disrupted the synthesis of photosynthetic pigments, inhibited photosynthesis and antioxidant enzyme activities, and hindered the growth and development.	(Wei et al., 2022)
Citrus reticulata Blanco	20 mM ZnSO_4_	Growth retardation; defoliation; reduction in photosynthesis, transpiration; reduced stomata size; disorganization of mitochondrial membranes and random distribution of cristae; phenols, sugars and starch contents decreased; oxidative stress	(Subba et al., 2014)
Hibiscus esculentus cv. Hassawi	20–40 mM ZnSO_4_	Induced lipid peroxidation, CAT, APOX, DHAR and GR activities; reduced non-enzymatic antioxidants	(Azooz, 2013)
*Phaseolus vulgaris* L.	0–550 µM ZnSO_4_	High Zn decreased macronutrient concentrations in all plant parts	(Havari Nejad et al., 2014)
*Lycopersicon esculentum*	0–250 mg kg^-1^soil ZnSO_4_. 7H_2_O	Decreased the various growth and length of the root and shoot, area of leaves and dry weight of root and shoot of tomato plants	(Vijayarengan and Mahalakshmi, 2013)
*Camellia sinensis*	0–30 µM ZnSO_4_	Zn-stress decreased net photosynthetic rate, transpiration rate, stomatal conductance, and content of chlorophylls *a* and *b*. Content of superoxide anion, malondialdehyde, hydrogen peroxide, and phenols, and electrolyte leakage were elevated in stressed plants.	(Mukhopadhyay et al., 2013)

## Mechanism of Zn uptake and long-distance transport under restricted Zn availability

Many genes are responsible for the complex sensing and make significant contributions that plants use to regulate the absorption and redistribution of micronutrients (Zhang et al., 2012; Ali et al., 2022b). The Zn^2+^ ions in the soil solution are transferred to the roots by mass flow and diffusion (Marschner, 2012). The amount of Zn in the rhizosphere (the area between the soil and the roots) is important for efficient uptake by plant roots (Khoshgoftarmanesh et al., 2018; Saleem et al., 2020c). Plants acquire majority of essential nutrients from the soil through their roots (Hassan et al., 2021; Sufyan et al., 2021; Hussain et al., 2022). But the obvious way for minerals to get into soil is through the physical and chemical breakdown of parent rocks. Most agricultural soils have Zn levels ranging from 10–300 µg Zn g^-1^ soil (Barber, 1995). The whole Zn in soil is not accessible to plants; its availability is determined by soil physicochemical qualities, the activity of plant roots and microorganisms in the rhizosphere, and other non-edaphic variables. In soil, Zn occurs as insoluble complexes or as adsorbed and exchangeable complexes (Noulas et al., 2018).

Plant roots assimilate Zn as the divalent cation. Moreover, organic-ligand-Zn complexes are also known to be taken up by plant roots. Zn is taken up by various transporter proteins, including ZIP family zinc-regulated transporter/iron-regulated transporter (ZRT/IRT), the heavy metal ATPase family protein (HMA), vacuolar iron transporter (VIT), natural resistance-associated macrophage protein (NRAMP), and the metal tolerance protein (MTP) family. The ZIP family promotes Zn entrance into the cytoplasm, HMA is involved in Zn efflux to the apoplast, and the MTP family enables Zn sequestration in cell organelles such as the vacuole (Gupta et al., 2016; Rudani et al., 2018). Multiple protein families, including ZRT/IRT-related protein (ZIP), HMA, and MTP, contribute to Zn transportation and regulation (Ricachenevsky et al., 2015), which is further elaborated in **Table 2**. Transporters affiliated with the VIT and NRAMP families have also been identified in Zn transportation (Karthika et al., 2018). The activity of various ZIP family members is governed by transcription factors from the basic leucine zipper (bZIP) family, which are linked to the Zn deficiency response element (ZDRE) of many genes that are susceptible to Zn deficiency. For example, in Arabidopsis ZIP1, ZIP3, ZIP4, ZIP5, ZIP9, ZIP10, and ZIP12, as well as IRT3, all these genes contain ZDRE, and their expression is regulated by bZIP19 and bZIP23 in the context of Zn deficiency (Verma et al., 2021). Zn^2+^ is transported from the soil *via* xylem loading with the assistance of specialized transporters located at the pericycle. The Zn^2+^ absorption from the soil solution is actively facilitated by root-specific membrane transporters. Plasma membrane transporters assist in crossing the plasma membrane and distributing it to the cytoplasm.

**Table 2:** Functions of zinc transporters-coding genes in plants.

Crop	Transport Family	Transporter name	Function	Reference
*Hordeum vulgare*	ZIP	*AtZIP1*	Increased Zn content in grains	(Tiong et al., 2014)
		*HvZIP7*	Increased Zn content in grains	
*Zea mays*		*ZmZIP3*	Uptake and translocation of Zn	(Li et al., 2015)
*Triticum dicoccoides*		*TdZIP1*	Transportation of Zn	(Durmaz et al., 2011)
*Oryza sativa*	P-type ATPase	*OsHMA2*	Root to shoot Zn translocation	(Yamaji and Ma, 2019)
		*OsHMA3*	Zn homeostasis	(Cai et al., 2019)
		*OsHMA7*	Zn homeostasis	(Kappara et al., 2018)
		*OsHMA9*	Zn homeostasis	(Lee et al., 2007)
	HMA	*OsHMA3*	Zn sequestration to root vacuoles	(Cai et al., 2019)
		*OsHMA2*	Root-to-shoot translocation of Zn.	(Yamaji et al., 2013)
*Triticum aestivum*		*TaHMA2*	Root-to-shoot translocation of Zn.	(Tan et al., 2013)
*Nicotiana tabacum*		*NtHMAα*	Root-to-shoot translocation of Zn	(Hermand et al., 2014)
		*NtHMAβ*		
*Arabidopsis thaliana*		*AtHMA2*		
		*AtHMA4*		

### Facilitated transport by channels

Zn is primarily absorbed by plants from soil through roots by a process of facilitated diffusion, which is mediated by membrane potential and transporters (Marschner, 2012). However, organic ligand-Zn complexes are sometimes also taken up by plant roots. Two possible physiological mechanisms are involved in the uptake, depending on the ligand secreted by plant roots. Therefore, mechanism I involves efflux of solvents, organic acids, and H^+^ ions, which improve absorption of Zn-complexes and release Zn^2+^ ions for absorption by root epidermal cells. Whereas, mechanism II entails the outflow of phyto-metallophores, which generate complex compounds with Zn and are then absorbed by root epidermal cells. This absorption process (i.e., method II) is, however, limited to cereal roots (Das and Green, 2016; Chatterjee et al., 2018). These mechanisms require the involvement of water molecules as solvents, in addition to variations in Zn content across the cellular membranes of roots. The primary driving force in the uptake of Zn^2+^ is the rapid polarization of the root plasma membrane, which is regulated by the action of the H^+^ ATPase. The H^+^ ATPase vigorously pumps H^+^ ions out of the cell, as a consequence of ATP hydrolysis. In the rhizosphere, H^+^ production enhances cation absorption by decreasing soil pH and hyperpolarizing the root plasma membrane (Kamaral et al., 2022). To transfer from one place to another inside a cell, divalent cations like Zn must be transported by particular transporter proteins (Eide, 2006; Lu et al., 2013; Gupta et al., 2016). These proteins are not closely related to ATP degradation, which supports passive transport rather than active Zn uptake.

### Active transport by transporters

After the Zn is taken up by plant roots, it is either stored there or transported to the upper parts (leaves and stems) of the plant. The majority of Zn in a plant is found in its roots, whereas a small percentage is transported to the leaves and stems (Sofo et al., 2018; Molnár et al., 2020). Numerous plant species-related variables are linked to Zn accumulation in roots or transport to the upper Xportion. Metal chelators and proteins that bind to metal are predominantly responsible for increased root Zn storage (Sofo et al., 2018). The metal binding proteins facilitate the absorption of Zn and metal ions by procuring the binding sites. Metals are often found to be stored in the vacuole, which is believed to be the primary source of metal requisitioning in root cells (Shabbir et al., 2020).

**Figure 2:**
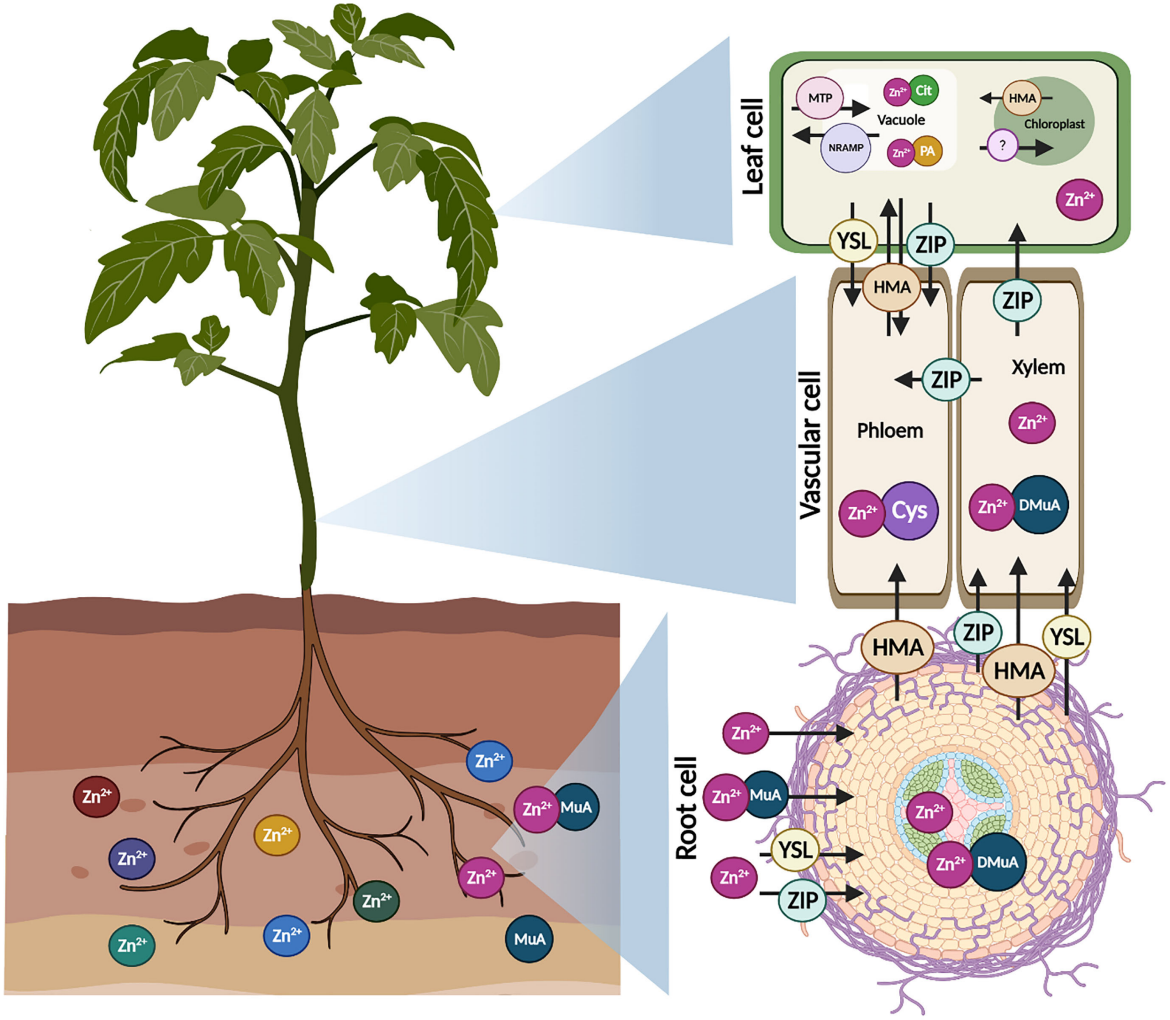
Mechanism of Zn transport in plants. Zn in soils penetrates in roots as Zn^2+^ or in a combination with mugineic acid (MuA), mediated by various transporter families such as yellow stripe like (YSL) and zinc regulated transporter (ZIP). Zn is either translocated as Zn^2+^ ion or as Zn complexes with MuA. Further, Zn interacts with 20-deoxymugineic acid (DMuA) promoting intercellular stability. Some transporters such as heavy metal ATPases (HMA), natural resistance-associated macrophage protein (NRAMP) and metal tolerance proteins (MTP) are present on the plasma membranes of cell and transport Zn to other parts of the plant cell. In phloem, Zn chelated with cysteine (Cys) participates in Zn sequestration. Chelated or free Zn^2+^ absorbed and mobilized in leaf vacuole and chloroplast. MTP transporter family required in the Zn sequestration. HMA and NRAMP transporters are capable of transferring accumulated Zn from leaf vacuole and chloroplast back into cytoplasm from loading into phloem during mobilization. Zn is chelated with citrate (Cit) and phytic acid (PA) in leaf vacuole.

Long-distance translocation (root to shoot) of Zn is mostly accomplished in the xylem *via* the transpiration stream (Page and Feller, 2015), as shown in **Figure 2**. Meanwhile, it requires energy and a number of active transporters are engaged in the transport and unloading of Zn in the xylem (Kamaral et al., 2022). Zn can flow as a free cation or as a compound in the xylem, although the acidic pH (5.5) of xylem sap promotes transportation as a neutral Zn^2+^ ion (Alves et al., 2004). Conversely, external conditions such as infection and seasonal climatic changes can alter pH in the xylem (Lu et al., 2013), offering more favorable circumstances for Zn chelation (Flis et al., 2016). The Zn^2+^ ions penetrate symplastically in live cells of the pericycle and xylem parenchyma, bordering the xylem after passing the boundary of the casparian strip in the root endodermis. In the xylem tissue, the continuous activity of H^+^ ATPase leads to hyperpolarization of the membrane, which in turn limits the efflux of positive ions from the cytosol. The movement of Zn^2+^ from the xylem cells to the apoplastic xylem is therefore an active transport (Alves et al., 2004). Therefore, specifically localized transporters (HMA family) on xylem cells facilitate the active efflux of symplastic Zn^2+^ ions (Sondergaard et al., 2004).

When Zn arrives at the phloem, it is transported to various plant parts and emerging sinks *via* short- and long-distance channels (Moreira et al., 2018). Furthermore, owing to the larger content of chelating agents like organic acids in phloem sap, Zn transport is relatively higher in phloem than in xylem (Verma et al., 2021). Zn is assumed to be carried through phloem tissues in ionic form or as Zn-complexes. Despite the fact that, xylem has a lower solute content, it is nonetheless vital in nutrition transport to numerous organs (Sohail et al., 2022). Phloem primarily nourishes emerging sink tissues such as grains, tubers, and so on (Page and Feller, 2015). Zn is transferred to the vacuole by a range of cation diffusion facilitators (CDFs), which are now typically referred to as MTPs (Sturikova et al., 2018). It is hypothesized that Zn root-shoot mobility is impeded by increased *MTP3* expression in the cortical and epidermal cells of roots. (Fujiwara et al., 2015) found that stimulation of *AtMTP1* improved Zn resistance in Arabidopsis *via* vacuole sequestration.

## Strategies to enhance Zn uptake

### Application of organic ligands

**Figure 3:**
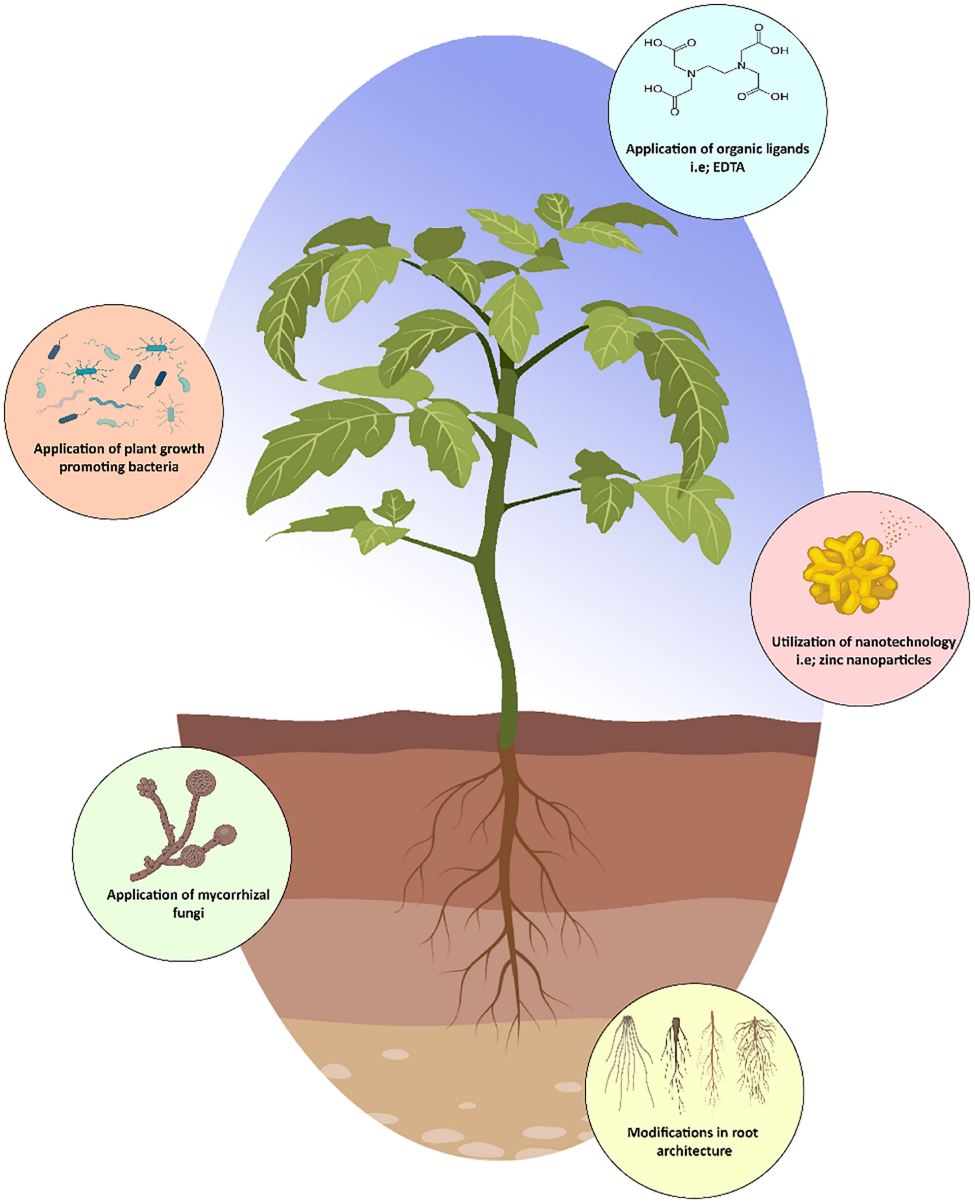
Strategies to enhance zinc uptake in plants.

Zinc is distributed biogeochemically in various basins. Overall, there are five distinct forms of Zn in soil: adsorbed, interchangeable, water-soluble, complex, and chelated **Figure 3**. The phyto-availability, leachability, and extractability of these Zn pools vary substantially (Alloway, 2008). The Zn levels in the soil, pH, soil texture, and the occurrence of competing cations all affect the presence of Zn in the abovementioned basins. However, the minerals, chemical structure, amount of organic matter, and pH of the soil all affect the availability of Zn (Wang et al., 2017). Zn availability is influenced by soil structure; sandy loam and organic soils are more prone to Zn deficiency than clayey or silty soils (Gong et al., 2020). Considering all aspects, soil pH has the greatest impact on the availability of Zn in soil. A higher pH soil has less availability of Zn than a lower pH soil (Sadeghzadeh, 2013). Various soil characteristics, including cation exchange ability, moisture content, and electrical conductivity, can influence the Zn availability in soil (Moreno-Lora and Delgado, 2020; Salinitro et al., 2020).

Utilization of organic ligands results in the formation of organic Zn compounds in soil, which may increase or reduce Zn availability in soil and plant uptake. Interestingly, (Piri et al., 2019) determined that humic acid treatment increased soil Zn adsorption capacities by 73–95%, whereas citric acid application lowered them by 52–68%. Similarly, (do Nascimento et al., 2020) demonstrated that citric acid treatment at 20 mmol/kg increased Zn adsorption in soil, hence increased Zn availability. Changes in pH of soil may also improve the effectiveness of organic ligands in enhancing Zn phyto-availability. Furthermore, it was revealed that citric acid (0.1 M) increased Zn absorption by plants up to 43% (Ke et al., 2020). It is documented that EDTA not only improves the availability of Zn in plants as well as in the soil up to 90%, consequently increasing straw and grain Zn levels by approximately 95% and 61%, respectively. Additionally, (Zhao et al., 2018) demonstrated more Zn absorption in wheat when treated with Zn-EDTA as compared to only Zn.

### Modifications in root traits

Root development influences the ability of a plant to obtain mineral nutrients from the soil. So, modification in the root architecture is necessary for acquiring Zn during particular circumstances of Zn deficiency. Furthermore, root development influences the ability of a plant to obtain mineral nutrients from the soil, e.g., increase the length, density of lateral root hairs, increase the active root surface area, and allowing the plant to explore greater soil volume, potentially improving Zn absorption efficiency (Verma et al., 2021). In barley, Zn deficiency was demonstrated to increase root length (Genc et al., 2007). Zn deficiency caused an elongation of the primary root systems of rice (Zeng et al., 2019). Meanwhile, Zn deficiency in Arabidopsis reduced primary root length while increasing the number and length of lateral roots (Jain et al., 2013). Azelaic acid induced 1 (AZI1), a member of the lipid protein family, was found to be essential for regulating primary root development in Arabidopsis under Zn deprivation; AZI1 knockdown mutants had smaller roots than wild-type plants (Bouain et al., 2018). AZI1 was suggested to be involved in the interaction between Zn deficiency and the defense system, because it is involved in the systemic resistance and in defensive signal. Moreover, it decreases root length in Arabidopsis plant in a Zn-dependent manner. Additionally, it has been proposed that the resistance to Zn shortage in rice is due to the fast growth of crown roots. A Zn shortage slows down the formation of crown roots. However, after being transplanted into a Zn-deficient environment, in comparison to Zn-inefficient rice genotypes, the Zn-efficient genotypes of rice generate new collar roots more quickly (Mori et al., 2016). Therefore, it may be advantageous for plants to acquire Zn through genetic variation of genes involved in modifying root architecture.

### Biostimulators

Plant biostimulators are an essential tool for contemporary agriculture as part of an integrated crop management system, aiding in the sustainability and resilience of agriculture (Hamid et al., 2021). This may be done in a number of ways, such as by supplying biostimulants or implementing a number of plant nutrition strategies, such as supplementing the food with trace elements that are typically lacking in the typical human diet (Yakhin et al., 2016). A biostimulant is a foreign substance or microbe, other than a fertilizer, that is administered to plants or the rhizosphere and promotes natural processes to increase plant nutritional efficiency (Rouphael and Colla, 2020). Biostimulants can be classified into a number of different groups, such as natural substances (extracts of seaweed, humic and folic acids, chitosan and vitamins, etc.), artificial substances (growth regulators and chelating ligands, etc.), inorganic compounds (non-essential but beneficial elements like Si and Se), beneficial rhizobacteria and fungi (du Jardin, 2015). As a result, biostimulants are progressively being introduced into production systems in order to influence physiological processes in plants in order to enhance production. On the contrary, certain biostimulants can also work as biofortification, increasing the mineral absorption and bioavailability for plants. The process of enhancing the nutritional value of staple food crops by agronomic techniques, such as the application of fertilizers to the foliar and soil, by adopting conventional and molecular breeding techniques, and the utilization of biotechnology, like genetic modification (Hawrylak-Nowak et al., 2019).

#### Mycorrhizal fungi

Arbuscular mycorrhizal fungi are one type of microbial biostimulant that is used in agricultural biofortification (Verma et al., 2021). These helpful soil fungi form mutualistic relationships with the majority of land plant species and create additional mycelia networks, which are useful for expanding the area of soil that has been examined and for facilitating the transport of both macro and micronutrients to plant tissues through incorporating mycorrhizal uptake by roots (Hamid et al., 2021). Mycorrhizal plants typically have higher nutrient concentrations and acquire secondary metabolites with both plant defense and human health-promoting activities than non-mycorrhizal plants because of the compensation mechanism by which the plant organic carbon is given to the fungal companion in exchange for mineral nutrients (Hart et al., 2015). Sustainable production and quality rely on the synergistic connection between Zn and arbuscular mycorrhizal fungi. Application of mycorrhizal fungi has been shown to improve grain Zn concentration in the field (Ercoli et al., 2017). In addition, it is seen that mycorrhizal fungi and a rise in Zn concentration work together to boost Zn nutrition (Al Mutairi et al., 2020). Hence, mycorrhizal plants has greater Zn content than non-mycorrhizal plants.

#### Plant growth promoting bacteria

Soil microbial diversity is thought to be crucial for ensuring that agricultural systems can continue to provide nutrition permanently. But the relationships between microbial diversity and environmental processes are not fully understood. The term “rhizosphere” is used to refer to the area where roots and soil come into contact and where the soil is changed by the roots. Roots have an effect on the microbial activity in the soil surrounding their rhizome. Plant growth-promoting bacteria are bacteria that colonize plant roots and have a positive influence on plant growth through either direct or indirect methods (Kumar et al., 2017). Therefore, increased nutrient availability to young plants under nutritional imbalance situations is achieved by plant growth promoting bacteria *via* modulating the solubility or absorption of nutrients (for example, by increasing the solubility of phosphorus, iron, and Zn). Biofortification with Zn-solubilizing plant growth promoting bacterial strains shows more promising effects after being injected with *Exiguobacterium auranticum* strain MS-ZT10, Zn and Fe levels increased by six-fold in *Triticum aestivum* grain (Shaikh and Saraf, 2017). Furthermore, the inoculation of strains *Bacillus halotolerant* (J143), *Enterobacter hormaechei* (J146), and *Pseudomonas frederiksbergensis* (J158) were drastically enhanced the wheat seed germination, plant development, and Zn absorption (Fahsi et al., 2021).

## Nanotechnology

The word “nanoparticle” is the foundation of nanotechnology and refers to a particle with a diameter of less than 100 nm. Size-dependent physicochemical features distinguish nanoparticles from their bulk or sub-micron/micron-sized equivalents (Sadeghzadeh, 2013; Imperiale et al., 2022). The main distinctive quality of nanoparticles is a comparatively higher surface area to volume ratio, which gives them a significant level of interactivity and physicochemical flexibility (Mauter et al., 2018). For example, nanoparticles are classified into several groups based on their structure and chemical composition, including oxides of metal, nano-polymers, quantum dots, and carbonaceous materials, with differing surface morphology such as particles, fibers, rods, and flowers (Dasgupta et al., 2017; Sudha et al., 2018). Because of their numerous qualities, they are employed in a variety of industries ranging from agriculture to manufacturing (Srivastava et al., 2018; Saleem et al., 2020b).

In agriculture, due to the unique properties (highly porous ratio, controlled-release kinetics to targeted sites) of nanomaterials, nanotechnology is used as nano-fertilizers. Nano-fertilizers may have been enclosed or bound with nanomaterial in order to regulate and limit the delivery of one or more nutrients to meet the critical nutritional requirements of a plant (Saleem et al., 2020a; Ahmed et al., 2021). Regarding the several types of nanoparticles, zinc oxide (ZnO) nanoparticles have demonstrated a significant impact on increasing plant growth and production. The ZnO nanoparticles provided Zn as an important nutrient for regulating fundamental plant processes, including germination, the synthesis of photosynthetic pigments, gene expression, cellular metabolism, and proliferation (Saleem et al., 2020c; Liu et al., 2022). Zn nanoparticles may be delivered either as foliar application or placed in the roots of plants, and both methods of application effectively transported the Zn due to their nano size and high surface area size ratio (Czyzowska and Barbasz, 2022). Recent findings by (Subbaiah et al., 2016) showed that spraying 25 nm ZnO nanoparticles on maize leaves had a favorable impact on plant development, yield, and Zn content in the grain. Interestingly, 100 ppm of ZnO nanoparticles were sprayed onto plants, and 36 ppm of Zn was found in the grains of those plants. Therefore, the higher Zn concentration may contribute to addressing the Zn deficiency in human and animal diets. The scientists came to the conclusion that the concentration of nanoparticles, the absorption of the particles, the capacity of plants to absorb the nutrient, and the size and transportation of the nanoparticles all affect the accumulation of Zn in different plant parts (Alsuwayyid et al., 2022). Consequently, ZnO nanoparticles can be used as an efficient nano-fertilizer to boost agricultural productivity, make it easier for plants to use micronutrients, and help to promote plant growth and development.

## Future perspectives

Zinc (Zn) is required for the metabolism of plants, enzyme function, and ion transport. The physicochemical involvement of Zn in soil-plant systems. Consequently, inadequate Zn availability in soil is a main consideration for plant nutrition, resulting in a significant loss in production and grain nutrient content. Several novel molecular breeding approaches are currently being used to improve Zn use efficiency in crops. Furthermore, potential biotechnological strategies, including Zn absorption and binding affinity by organic acids, as well as enhancing Zn absorption by transporters, assist in increasing Zn uptake and efficiency in plants, thereby overcoming dietary Zn deficiency. The ZIP family enables Zn to enter the cytosol, the HMA family aids in Zn efflux to the apoplast, and the MTP family aids in Zn sequestration in cellular compartments such as the vacuole. During transpiration, long-distance root-to-shoot transfer occurs in the xylem. Additionally, the direct application of organic ligands improves Zn absorption by plants. In soil, organic ligands produce Zn complexes that can enhance or limit the Zn availability and absorption of plants. Therefore, Zn transporters play an important role in sustaining Zn uptake during Zn deficiency circumstances. As a result, studying these transporters and their regulatory mechanisms can help us better understand the process of Zn uptake and transport in plants. Despite the fact that remodeling the architecture of the root system improves the acquisition of Zn under Zn deficiency. Moreover, the plant growth promoting bacteria increase the accessibility of nutrients to developing plants with nutritional deficiencies by increasing the solubility of Zn. Various plant growth-promoting microorganisms improve nutrient availability in nutrient-deficient environments. Besides, nanoparticles have separate biophysical characteristics from mass-sized or micron-sized materials. The high surface-to-volume ratio of nanoparticles confers strong reactivity and physicochemical flexibility, which promotes agricultural productivity by increasing the utilization of micronutrients. Thus, the greater understanding of the molecular regulatory network involved in the regulation of Zn deficiency response and Zn homeostasis would contribute to Zn phyto-availability by developing crops with an increased content of bioavailable Zn in plants, which has been considered as most cost-effective methods for combating Zn deficiency in plants and malnutrition problem in humans.

## Authors contributions

MS, KU and MA conceived and designed the article and HJ critically revised the manuscript and approved the final version. MS and KU, MA wrote the manuscript. MR and HJ critically edited and revised the manuscript. All authors contributed to the article and approved the submitted version.

## Funding

This work was supported by the Qatar University vegetable factory project QUEX-CASMJF-VF-18-19.

## Acknowledgments

Open Access funding provided by the Qatar National Library.

## Conflict of interest

The authors declare that the research was conducted in the absence of any commercial or financial relationships that could be construed as a potential conflict of interest.

## Publisher’s note

All claims expressed in this article are solely those of the authors and do not necessarily represent those of their affiliated organizations, or those of the publisher, the editors and the reviewers. Any product that may be evaluated in this article, or claim that may be made by its manufacturer, is not guaranteed or endorsed by the publisher.
